# Chest X-ray dataset and ground truth for lung segmentation

**DOI:** 10.1016/j.dib.2023.109640

**Published:** 2023-10-05

**Authors:** Rima Tri Wahyuningrum, Indah Yunita, Achmad Bauravindah, Indah Agustien Siradjuddin, Budi Dwi Satoto, Amillia Kartika Sari, Anggraini Dwi Sensusiati

**Affiliations:** a Department of Informatics Engineering, Faculty of Engineering, University of Trunojoyo Madura, Indonesia; b Department of Health, Faculty of Vocational Studies, Universitas Airlangga, Indonesia; c Department of Radiology, Medical Faculty, Universitas Airlangga Hospital, Indonesia

**Keywords:** Chest X-ray, Covid-19, Medical image segmentation, Deep learning, Diagnosis

## Abstract

Chest X-ray images are a valuable tool for accurately and efficiently diagnosing Covid-19 with the assistance of computer technology. These images enable the detection of diseases in internal organs, particularly the lungs, by providing crucial information about the pathological state of the lungs and other internal organs and tissues. Segmentation plays an essential role in the earliest stages of disease detection through computer-assisted analysis of medical images. This method enables the extraction of significant elements from the image, facilitating the identification of relevant areas. In the subsequent stage, healthcare professionals might acquire more precise diagnosis outcomes. Deep learning plays a significant role in developing models to achieve exact and efficient diagnostic results in picture segmentation and image classification procedures. However, using deep learning models in the image segmentation process necessitates the availability of image datasets and ground truth that radiologists have validated to facilitate the training process. The dataset provided in this article comprises 292 chest X-ray images obtained from Airlangga University Hospital in Indonesia. These images are accompanied with ground truth data that has been meticulously verified by radiologists. The offered X-ray images encompass those of patients diagnosed with Covid-19, pneumonia and those representing normal conditions. The provided dataset exhibits potential utility in advancing artificial intelligence techniques for segmentation and classification procedures.

Specifications Table

**Table:** 

Subject	Biomedical Engineering
Specific subject area	Medical Image Processing, Lung Segmentation, Pulmonary Disease, Covid-19,
Type of data	Image
How the data were acquired	Chest X-Ray data from Airlangga University in DICOM format was converted into .bmp format with a resolution of 256 × 256 pixels using python and segmented using U-Net deep learning model which had previously been trained using Chest X-Ray data and ground truth from Qatar University. Then the segmentation results are checked and validated by radiologists and used as ground truth for the dataset.
Data format	Analyzed
Description of data collection	The dataset consists of 292 chest X-ray images and their ground truths that have been validated by radiologists. There were 207 Chest X-Ray images of covid patients, 53 X-Ray images of pneumonia patients, and 32 X-Ray images of normal patients. Dataset and ground truth are in .bmp format with a resolution of 256 × 256 pixels.
Data source location	Airlangga University Hospital Surabaya Indonesia
Data accessibility	Repository name: Mendeley Data Data identification number: 10.17632/2jg8vfdmpm.1 Direct URL to data: https://data.mendeley.com/datasets/2jg8vfdmpm/1
Related research article	R. T. Wahyuningrum, I. Yunita, I. A. Siradjuddin, B. D. Satoto, A. K. Sari, and A. D. Sensusiati, “Improvement of chest X-ray image segmentation accuracy based on FCA- Net,” *Cogent Eng.*, vol. 10, no. 1, 2023, doi: 10.1080/23311916.2023.2229571

## 1 Value of the Data

• Chest X-ray images in this dataset can be used for image processing using artificial intelligence models, especially in segmenting lung images. Because in this dataset, there is ground truth that radiologists have validated.
• Artificial intelligence researchers can benefit by using this dataset because it can be used to experiment with the artificial intelligence architecture models they have designed. In addition, medical professionals can also help from deep learning models that have been trained using this dataset so that diagnosing lung disease becomes faster, more accurate, and more efficient.
• This dataset can be used for artificial intelligence-based research using deep learning models for lung segmentation and diagnosis of Covid-19 disease.

## 2 Objective

Chest X-ray images are an essential part of the procedure for diagnosing diseases that involve visual representations of human organs. Accurate interpretation of information is a significant challenge for radiologists as a first step in diagnosing disease. Therefore, the computer-assisted diagnosis process can be used as an alternative for the disease diagnosis process more quickly, accurately, and efficiently. Image segmentation is an essential stage in computer vision to help the disease detection process become more accurate because this segmentation process can represent important features of X-ray images. In the segmentation process, deep learning models become one of the approach methods with better performance and can achieve high accuracy [1]. However, datasets and ground truth are needed to train deep learning models for image segmentation. The dataset available in this manuscript can be used in the deep learning model training process because it already has ground truth validated by the radiologist.

## 3 Data Description

The dataset consists of 2 main folders available in the data repository, namely “Data Chest X-ray RSUA (Annotated)” and “Data Chest X-Ray RSUA (Validated).” The folder “Data Chest X-ray RSUA (Annotated)” contains data from Airlangga University Hospital, which has been converted into .bmp format. The dataset in the folder has also been given a line annotation that follows the form of the segmentation results and is then shown to the radiologist for validation. The folder “RSUA (Validated) Chest X-Ray Data” contains chest X-ray image data and segmentation or ground truth results that radiologists have validated.

The data containing validated ground truth totaled 292 images consisting of 207 X-ray images of COVID-19 patients, 53 X-ray images of pneumonia patients, and 32 of normal patients. Each image is in .bmp format with a 256 × 256 pixels resolution.

**Fig. 1:**
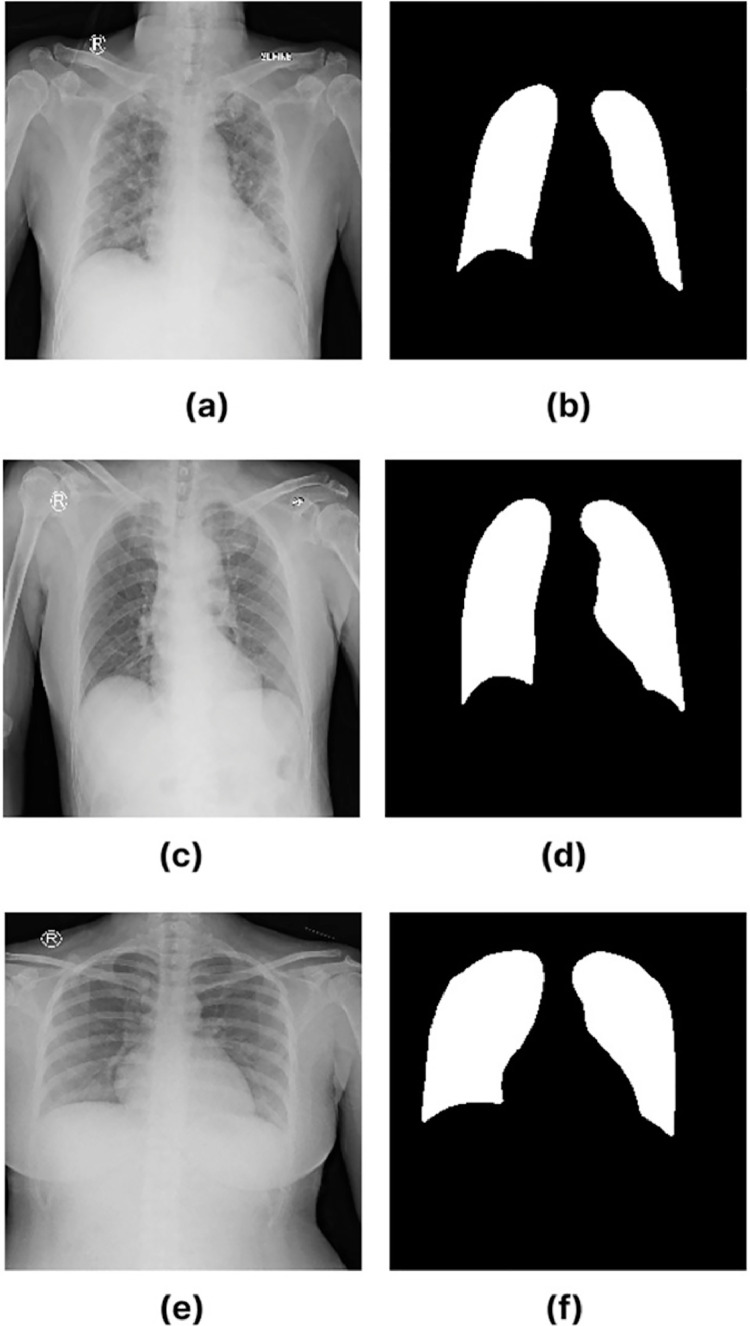
Covid-19 chest X-ray image (a), Covid-19 ground truth (b), Pneumonia chest X-ray image (c), Pneumonia ground truth (d), Normal chest X-ray image (e), Normal ground truth (f).

In the data repository, image files have been converted into .npy format using Python.This .npy format file contains the pixel matrix values of the image. This file can be used to perform image processing more lightly. Fig. 1 shows an example of a chest x-ray image and its segmentation results for each class (Covid-19, Pneumonia and normal).

## 4 Experimental Design, Materials, and Methods

**Fig. 2:**
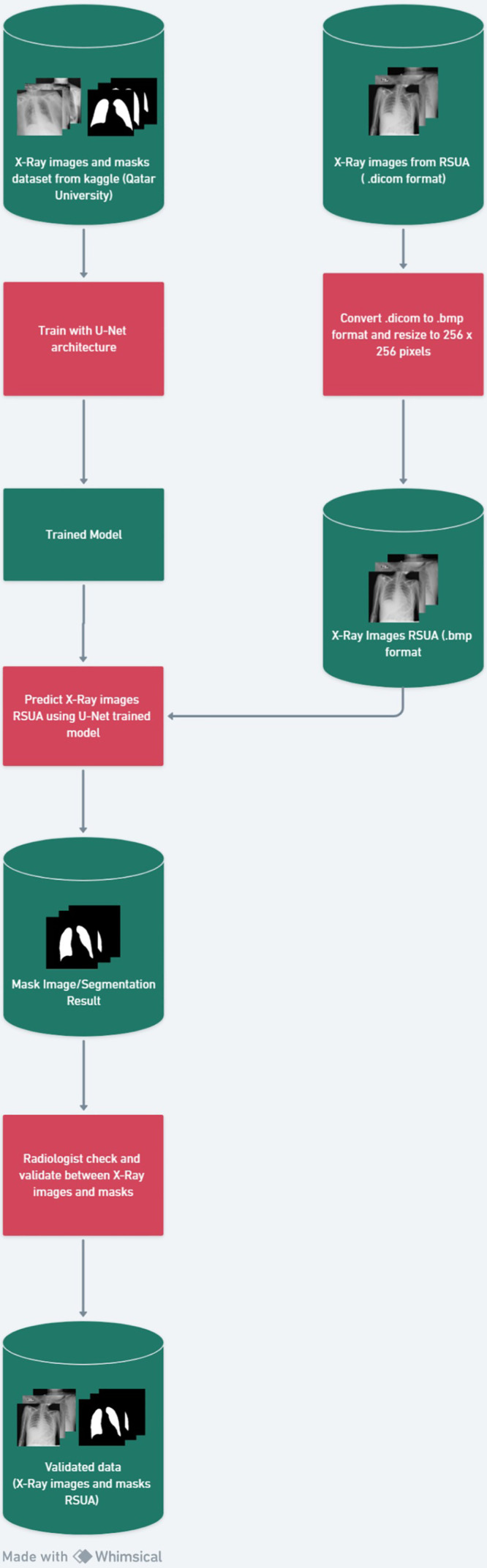
Data curation process.

Fig. 2 shows the data curation process. U-Net model was trained using chest X-ray datasets derived from other repository sources, namely datasets from Qatar University, which already have ground truth with a total of 1500 data of 500 covid-19, pneumonia, and normal images in each class. The accuracy level produced by this model is DSC (Dice Similarity Coefficient) of 97 % and IoU (Intersection over Union) of 94 %. This model will later be used to segment the chest X-ray image from Universitas Airlangga Hospital because the dataset provided by the hospital still needs to have ground truth.

The chest X-ray image dataset from Universitas Airlangga Hospital amounted to 657 images in DICOM format, which were then converted into .bmp format using Python programming. The images that were successfully converted into .bmp format amounted to 371 images consisting of 273 images infected with covid-19, 59 images infected with pneumonia, and 39 normal images.

Images that have been converted into .bmp format are then predicted using the U-Net model that has been created to produce segmented images. Furthermore, line annotation is made following image segmentation results and overlaps on the chest X-ray image according to the radiologist's request to be shown to the radiologist and validated.

**Fig. 3:**
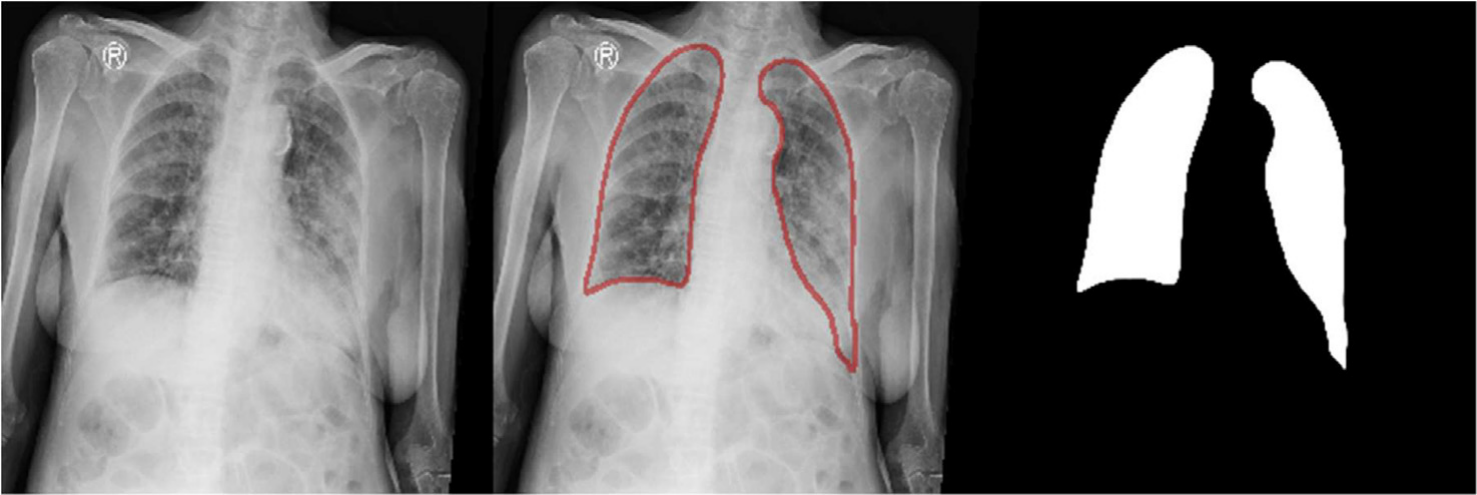
Merge of chest X-ray Image, Line annotation image, and segmentation image that shown to radiologist.

From the 371 images submitted to radiologists, there were 292 segmented images validated to be accurate by radiologists, consisting of 207 images infected with Covid-19, 53 images infected with pneumonia, and 32 images normal. These validated images are then collected and stored in a dataset repository. Fig. 3 shows an example of a merged chest X-ray image, line annotation, and radiologically validated segmentation results.

## Ethics Statements

This research involves humans and has passed ethical approval from Universitas Airlangga Hospital with letter number 093/KEP/2022. In this letter is stated that The Research Ethics Committee of Rumah Sakit Universitas Airlangga with regards of protection of human rights and welfare of research subjects, has carefully reviewed the research protocol with this dataset.

## CRediT authorship contribution statement

**Rima Tri Wahyuningrum:** Formal analysis, Writing – review & editing, Supervision. **Indah Yunita:** Software, Writing – original draft. **Achmad Bauravindah:** Data curation, Software. **Indah Agustien Siradjuddin:** Project administration, Visualization. **Budi Dwi Satoto:** Conceptualization, Methodology, Writing – review & editing. **Amillia Kartika Sari:** Investigation, Validation. **Anggraini Dwi Sensusiati:** Supervision.

## Data Availability

RSUA Chest X-Ray Dataset (Original data) (Mendeley Data)

## References

[1] Wahyuningrum R.T., Yunita I., Siradjuddin I.A., Satoto B.D., Sari A.K., Sensusiati A.D. (2023). Improvement of chest X-ray image segmentation accuracy based on FCA-Net. Cogent. Eng..

